# How Long to Sleep

**Published:** 1890-04

**Authors:** 


### HOW LONG TO SLEEP.

The popular belief that men of extraordinary mental activity are, as a rule, light sleepers, is not justified by the facts. What the just and right allowance of sleep may be for the individual does not seem to depend altogether upon the amount of mental or physicial work done, and is, to a considerable extent, determined by inheritance and idiosyncrasy. The idler and the pleasure lover often seem to sleep longer and more easily than the laborious brain worker, and cases may be traced where a tendency to light or heavy sleep has run strongly in families, irrespectively of the occupations, or of the physical and intellectual activities of individuals. The only safe guide in determining such a question is experience. If good health and full intellectual efficiency can be preserved by six hours' sleep, there seems no motive for making efforts, probably destined to failure, to secure eight hours. But care should be exercised that short sleep has not been the result merely of a long continued bad habit, and that every opportunity is afforded to the organism to procure that amount of sleep that seems normal for it. Hence moderately early hours, quiet, freedom from sources of disturbance are necessary, and for a prolonged period, before we can feel sure that the amount of sleep that seems natural to us is really so. Eight hours has been fixed by general consent as the happy mean, and we have no objection to make to it, although it is, perhaps, a liberal allowance for adults in vigorous health. The young and the ailing may with advantage take more, and, indeed, can hardly have too much of so excellent a tonic and restorative as sleep.

Apart from such causes of insomnia as profound sorrow or harrowing anxiety, rational treatment of sleeplessness can do much. The condition of bed and bedroom should be inquired into, and modified as common sense rules may dictate. The occupations of the sufferer, both during the day, and more especially during the hour preceding bedtime, should be carefully inquired into. Most authorities, wisely, no doubt, recommend that the bad sleeper should break off his daily routine some time before the hour of retiring to rest ; that he should try the distracting influence of conversation, a stroll, a novel, or a cigar, and that every effort should be made to prevent the overtaxed brain from pursuing during the night the well beaten track of the day. A tepid bath at bed-time often acts well, whereas a very hot or cold bath is to many persons injurious. The bed-clothes should combine comfortable warmth with lightness and ventilation, and the head should be placed somewhat high. There is a widespread impression that it is easier to sleep with the head to the north than in any other position ; but it is difficult to see any valid ground for this idea. The various plans that have been devised of striving to cheat the mind into forgetfulness of self, such as counting numbers, picturing flowing water, reckoning up an imaginary flock of sheep, do not in general seem to be very successful. We must bear in mind, above all things, that sleep is exceedingly automatic, and that if we strive to make it purposive we defeat ourselves. The use of narcotics cannot be advantageously defined in general terms ; but there can be little doubt that vast injury is daily wrought by the unwise use of chloral, bromides, and opium ; whereas their careful employment, especially in severe disease and in acute insomnia, may be productive of immense benefit.

